# Single cell metrics of the efficacy of CAR T cells

**DOI:** 10.1186/2051-1426-3-S2-P324

**Published:** 2015-11-04

**Authors:** Gabrielle Romain, Harjeet Singh, Ivan Liadi, Jay R Adolacion, Badrinath Roysam, Laurence Cooper, Navin Varadarajan

**Affiliations:** 1 University of Houston, Houston, TX, USA; 2 University of Texas MD Anderson Cancer Center, Houston, TX, USA; 3 University of Houston, University of the Philippines, Houston, TX, USA

## 

T cells genetically modified to enforce expression of a chimeric antigen receptor (CAR) have shown considerable promise in clinical trials even in tumors refractory to all other treatment methods. In particular, the use of CAR T cells rendered specific for CD19 demonstrated significant anti-tumor effects in patients with CD19^+^ chronic lymphocytic leukemia (CLL) and acute lymphoblastic leukemia (ALL). Despite the clinical promise of CAR therapy in achieving complete responses, their efficacy remains unpredictable and new approaches are needed to address this question and *a priori* define the therapeutic potential of T cell based therapies. An integration of the existing clinical data suggests that the efficacy of ACT is likely balanced by two requirements of the T cell infusion product: immediate cytotoxicity to enable eradication of the tumor and the ability to persist and establish memory to ensure long term benefit.

Using our novel single cell multiparametric assay, Timelapse Imaging Microscopy In Nanowell Grids (TIMING), we compared the efficacy of two second generation (CD28 endodomain) CD19 specific CAR constructs, bearing CD8a and IgG4 hinge respectively, by tracking their interaction with NALM6 tumor cells *in vitro*. Although there were no discernible differences using a population level assay, we demonstrate using TIMING that significantly more CAR T cells bearing the CD8a hinge participate in serial killing. This superior efficacy was confirmed in a mouse model in which CART cells containing the CD8a extracellular domain were superior in controlling the disease.

In order to identify biomarkers which distinguish between killer (and serial killer) CAR T cells and non-killers, we performed single cell multiplexed gene expression profiling (96 genes) subsequent to a TIMING assay. Across three separate donors, killer CAR T cells consistently expressed higher transcript levels of the cytotoxic molecule Granzyme B, the costimulatory receptor CD137 (41BB) and the regulatory receptor TIM3 (HAVCR2).

By utilizing multicolor flow cytometry, we confirmed that CD137 was overexpressed in cytotoxic CAR T cells and that its stimulation was associated with higher cytotoxicity and lower expression level of the immunoregulatory receptors CTLA4 and PD1. By comparison, TIM3 was not enriched in killer CAR T cells but rather was a likely marker for effector status since blocking TIM3 boosted cytotoxic responses.

In aggregate, these results demonstrate the utility of our TIMING single cell methodology in uncovering not only the dynamic profile of T cell behavior but in also uncovering the phenotypic biomarkers of CAR T cells with superior functional efficacy.

